# Impact of matrix support on older adults in primary care: randomized community trial

**DOI:** 10.11606/s1518-8787.2021055002685

**Published:** 2021-04-05

**Authors:** Luciana Colares Maia, Thomaz de Figueiredo Braga Colares, Edgar Nunes de Morais, Simone de Melo Costa, Antônio Prates Caldeira

**Affiliations:** I Universidade Estadual de Montes Claros Centro Mais Vida Eny Faria de Oliveira Departamento de Clínica Médica Montes ClarosMG Brasil Universidade Estadual de Montes Claros. Centro Mais Vida Eny Faria de Oliveira. Departamento de Clínica Médica. Montes Claros, MG, Brasil; II Universidade Estadual de Montes Claros Centro Mais Vida Eny Faria de Oliveira Montes ClarosMG Brasil Universidade Estadual de Montes Claros. Centro Mais Vida Eny Faria de Oliveira. Montes Claros. MG, Brasil; III Universidade Federal de Minas Gerais Instituto Jenny Faria de Oliveira Núcleo de Geriatria e Gerontologia Belo HorizonteMG Brasil Universidade Federal de Minas Gerais. Instituto Jenny Faria de Oliveira. Núcleo de Geriatria e Gerontologia. Belo Horizonte, MG, Brasil; IV Universidade Estadual de Montes Claros Centro de Ciências Biológicas e da Saúde Departamento de Odontologia Montes ClarosMG Brasil Universidade Estadual de Montes Claros. Centro de Ciências Biológicas e da Saúde. Departamento de Odontologia. Montes Claros, MG, Brasil; V Universidade Estadual de Montes Claros Centro de Ciências Biológicas e da Saúde Departamento de Saúde da Mulher e da Criança Montes ClarosMG Brasil Universidade Estadual de Montes Claros. Centro de Ciências Biológicas e da Saúde. Departamento de Saúde da Mulher e da Criança. Montes Claros, MG, Brasil

**Keywords:** Health of the Elderly, Matrix Support, Primary Health Care, Public Health, Clinical Trial

## Abstract

**OBJECTIVE:**

To analyze the effect of matrix support on health for older adults in primary care according to the dimensions of frailty measured with the Clinical-Functional Vulnerability Index-20 (IVCF-20).

**METHODS:**

This is a randomized controlled community trial, developed in the Northern Minas Gerais state, Brazil, in 2018. Initially, the stratification of clinical and functional vulnerability of older adults supported by six Family Health Strategy teams occurred with the IVCF-20. Subsequently, three teams were drawn to receive matrix support for six months, and the others for control. In this intervention, face-to-face educational activities were developed for health teams. Descriptive statistics were performed, followed by bivariate analysis by Pearson’s chi-square test, to compare the variables of the IVCF-20 between the two moments (before and after the intervention), with a 5% significance level. Relative risks and respective 95% confidence intervals (95%CI) were estimated.

**RESULTS:**

The groups were similar before intervention, and the effect of matrix actions was positive for most dimensions measured by IVCF-20 (instrumental daily living activity, cognition, mood, mobility, communication, and multiple comorbidities). At the end of the research, the percentage of frailty in the group assisted by professionals participating in matrix support was lower than that of the control group.

**CONCLUSIONS:**

Matrix support actions, such as pedagogical attribution and horizontal care for health teams, have the potential to contribute to the articulation of models of care for older adults.

**REBEC:**

registro BR-7b9xff

## INTRODUCTION

The fast aging of the Brazilian population defines a demographic and epidemiological profile with increasing and requiring demands on the health system, making it necessary to articulate tools for the organization, structuring, and integration of the different points of care for older adults^1^. This phenomenon requires a care model that integrally addresses older adults, in accordance with the principles and guidelines of the Brazilian Unified Health System (SUS)^1,2,5^. The great challenge is to reshape the fragmented health system in a health care network that responds in a timely, continuous, and integrated manner, based on demographic and epidemiological aspects of the population^6,7^.

The ideal structure of health care should direct the existing flow and services in care networks, acting cooperatively, interdependently, and coordinately with the Primary Health Care (PHC)^1,2,7,8^. In this context, the Family Health Strategy (FHS) – which is the priority form of PHC organization in the country – is highlighted in the operationalization of the care network for older adults^1,7^. Comprehensive care presupposes identifying the most vulnerable, as well as monitoring the clinical-functional evolution and workflow in the organized network^9^. This would result in fewer medical appointments and hospitalizations and better management of functionality, in addition to better cost/effectiveness in the health system^1,6,7,10,11,13,14^.

There are good experiences of PHC strengthening that combine the empowerment of health teams with support for the management of health care, such as matrix support^17,18^. Matrix support (MS) in health emerged as an innovative strategy, based on the promotion of an organizational system of continuous care and educational services network within the scope of SUS^17^. The main idea of the MS is to operationalize the integrated functioning of the network by ecoparticipative and interprofessional relationship of its elements^17,19-21^. Matrix support consists of theoretical and conceptual reconstruction of interdisciplinary work methodology in health — considering the relationship between reference teams (RT) and matrix support professionals (secondary care). This support depends on the relationship between health teams, the extension of specialized care scenarios, and the shared elaboration between professionals of the reference team and specialists who offer the MS, clinical and sanitary guidelines^20^. Therefore, the constitutive elements of the MS involve services that compose the SUS network and relationships formed between teams to operationalize this support.

Among the successful experiences of MS, the case of mental health can be highlighted, with a reform model of care in shared interprofessional practices associated with co-responsibility between team and users, contributing to the development of actions to decentralize care^17,18^. The proposal of a matrix model in the health care of older adults is an idea not yet consolidated in the country and it is in line with the needs of health services to enhance care network^1,3,8^.

The referral to the different levels of the health care network of the older adult varies according to the degree of vulnerability identified (fragile and non-fragile)^1,7,10,12,15,16,22^. For the screening of clinical-functional strata in this population, there are several instruments^15,16,22,26^, including the clinical-functional vulnerability index (IVCF-20), which was developed and validated in Brazil and it has a good correlation with other instruments used internationally ^15,16,26,27^. Due to the ease of application, the IVCF-20 is a good tool for the FHS teams in the evaluation and follow-up of older adults^15,16^. This study analyzes the effect of matrix support on health to older adults assisted by the FHS based on the level of frailty measurement, according to the IVCF-20.

## METHODS

This is a randomized controlled community trial (RCT), conducted in the municipality of Montes Claros, in the Southeast region of Brazil, in 2018. At the time of the study, the urban population was approximately 400,000 inhabitants and it recorded more than 80% coverage by the family health strategy, with 121 teams. Considering the need for contexts with low turnover among health professionals, the research was developed in scope centers of the Medical Residency Program in Family and Community Medicine (PRMMFC – *Programa de Residência Médica em Medicina de Família e Comunidade*). During this period, the municipality had 12 regional centers, each composed of three teams, and each team was responsible for five micro-areas. Two poles were drawn, between the 12 centers, for immediate intervention, and two poles for control group, using the random number generator program (randomnumbergeneration.intemodino.com). The poles drawn presented similarities in relation to the composition of the teams, they were areas of coverage of the PRMMFC and presented sociodemographic similarity. The definition of teams for intervention or control was randomized by simple draw. The intervention unit was the health team, and the outcome was measured in the older population assisted by the respective teams.

For the sample estimation of the number of older adults followed in each group, the type 1 error (alpha) of 5% and a 80% power of the study in a two-tailed test were considered. The clinical-functional vulnerability rate (frailty) of 25% was considered and, after matrix support, 10%, that is reduction of 15% occurred. Thus, the proposed sample was defined in at least 150 older adults in each study group: intervention and control.

Based on the random selection of health teams, the inclusion of older adults was also performed randomly, based on the relationship of people aged 60 years or older assisted by the teams of the Residence of Family and Community Medicine, respecting eligibility criteria for the study. Older adults able to answer the questionnaires and, when disabled, those older adults who had caregivers/guardians available during the visit to provide the requested information participated in the study. The data collection was performed at home, in the morning, evening or night shifts and on all days of the week, preferably with prior scheduling. Older adults not located in at least three visits, on different days and times, were replaced by draw.

The Consort diagram (Consolidated Standards of Reporting Trials) (Figure 1) shows the details of inclusion, allocation, and follow-up of the study participants, in the intervention group (with matrix support to the FHS) and in the control group (without matrix support to the FHS). The 402 participants eligible to compose the sample of older adults were distributed in: 197 in the intervention group (IG) and 205 in the control group (CG). The higher number of participants in relation to the sample estimation was included considering the possibility of losses, which did not occur during the study period.

**Figure:**
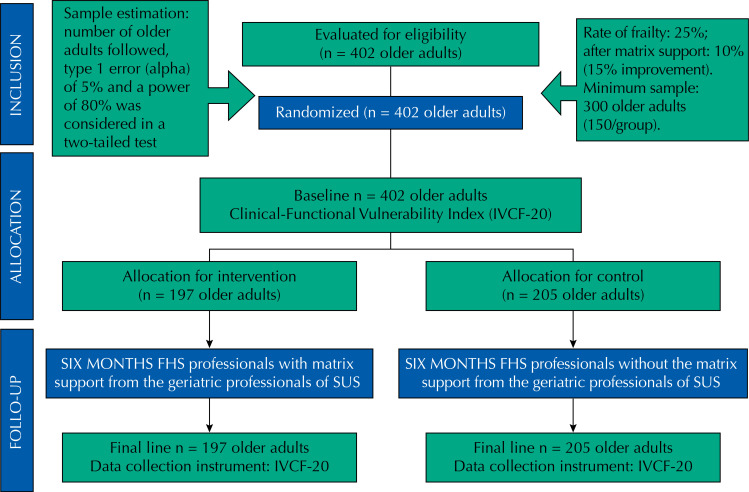
Consort diagram of allocation, follow-up, and analysis of individuals of the matrix support in older adults’ health, Montes Claros, Minas Gerais, Brazil.

The selected older adults answered the IVCF-20, a screening questionnaire with multidimensional character, rapid application (five to 10 minutes) and easy interpretation of the result, which can be performed by all the health team, that probably contributed to positive results in the context of recognition and multiprofessional management of frailty in PHC^15,16,26^.

This instrument includes domains considered predictors of clinical and functional vulnerability and adverse outcomes, such as functional decline and death in the older adults^15,16,26,27^. Moreover, these instruments considers the following dimensions: self-perceived health, activities of daily living (ADL), cognition, mood, mobility, communication, and multiple comorbidities. The IVCF-20 provides the creation of a score that defines the risk of frailty. The final score to stratify frailty in this study was dichotomized in fragile (score ≥15) and non-fragile (score < 15)^15,16,22,26,27^. In addition to the IVCF-20, information on age, gender, and income of the older adults was collected.

Data collection was performed by a specially trained team composed of nursing professionals and medical students. Prior to the beginning of the research, a pilot project was conducted, in a different area, for the calibration of the interviewers. Data collection occurred before (baseline) and six months after the end of the intervention (final line) by the training of the FHS professionals to support the older adults’ matrix health support. The interviewers were the same, both in the pre-intervention and in the post-intervention, and they were not aware of which health teams would be under intervention or control.

The training of health teams was carried out during six months in the PRMMFC centers, with monthly meetings coordinated by a geriatric physician working in the SUS. A modular training schedule was established based on the structuring axes of the older adults health^7^, without affecting the service of the health units selected for intervention. The team was called in advance by the Municipal Health Department and, the by phone, they received a confirmation of the meeting by the person in charge of the MS. Professionals who were members of each pole participated in the educational activities: three from medicine, six from nursing, and 15 community health agents. All professionals from the categories described above, linked to the selected poles, were considered eligible for participation in the intervention. The frequency of participation was 100%.

This proposal was based on the way of mental health matrix support, already carried out throughout the municipality PHC. Thus, each monthly meeting lasted four hours, based on tutorial actions, dialogued class, discussion of clinical cases, as well as care and construction of therapeutic plans in group. It is emphasized that the educational and care planning of the meetings valued the teams’ demands, based on discussion of clinical cases of the older adults with clinical-functional stratification, followed by the elaboration and shared monitoring of care plans, with the management of the user’s health by PHC teams.

At first, a survey was conducted on the main problems presented by the older adults in the IG: forgetfulness, falls, loss of functionality, polypharmacy, iatrogenic, lack of family support, and urinary incontinence. The themes aggregated structuring axes of integral care of the older adults (giants of geriatrics)^7^– 1) frailty syndrome; 2) cognitive disability; 3) postural instability, falls, and immobility; 4) iatrogenic; and 5) incontinence – topics that compose the IVCF-20 dimensions^27^. Thus, the monthly programming of the matrix experience in health of older adults was carried out, regarding the demands of the teams. We emphasize that the e-mail and telephone number of the tutor was available to the IG.

Descriptive statistics were performed, followed by bivariate analysis by Pearson’s chi-square test to compare the variables of IVCF-20 before and after the intervention, assuming a 5% significance level. The relative risk (RR) was estimated for the general classification of the frailty index based on the final scores of the instrument (categorized into frail and non-frail) and it was also estimated for the variables that compose the IVCF-20 dimensions. Data were analyzed with the IBM^®^ SPSS^®^*software* (version 22.0).

This study was conducted in accordance with Resolution 466/12 of the National Health Council, Brazilian Ministry of Health, opinion No. 1,628,652. It was also registered on the Brazilian Clinical Trials Registration Platform (ReBEC *–Plataforma de Registro Brasileiro de Ensaios Clínicos*)^a^, under registration number BR-7b9xff.

All participants were duly informed about the research and presented their consent by signing the free and informed consent form. For those who could not sign, a collection of fingerprints was performed in a specific area of the consent form. Anonymity and confidentiality of the information provided were ensured, this information was exclusively used for research purposes.

We emphasize that, with the completion of post-intervention data collection, the other health teams received the same educational intervention. Throughout the research process, the older adults classified with a high degree of frailty were referred for specific follow-up (secondary care). During the study, the older adults of the CG continued to be assisted and monitored by the FHS teams to which they were enrolled, using the usual resources available in the municipality.

## RESULTS

Table 1 presents the comparison of some characteristics of IG and CG in the baseline of the study. It was noticed that most older adults were female, aged between 60 and 74 years and with income of up to two minimum wages in force during the period of data collection. These variables were defined for the analysis of comparability of the groups, not registering statistically significant differences between demographic and economic variables (gender, age, and income) of the groups, in the baseline of the study.

**Table 1:** Demographic and economic characteristics of the intervention (IG) and control (CG) groups, in the baseline of the study, Montes Claros, Minas Gerais, Brazil, 2018.

Characteristic	Intervention (IG)	Control (CG)	P
	n (%)	n (%)	
Sex			0.224
Female	126 (63.9)	138 (67.3)	
Male	71 (36.1)	67 (32.7)	
Age (years)			0.916
60–74	118 (58.3)	118 (57.6)	
75–84	56 (29.4)	62 (30.2)	
≥ 85	23 (12.3)	25 (12.2)	
Income (in minimum wages)^a^			0.202
> 2	74 (42.8)	91 (46.7)	
≤ 2	109 (57.2)	104 (53.3)	

^a^ Minimum wage in force in 2018 (R$ 937.00).

Table 2 presents the stratification of the older adults regarding clinical and functional vulnerability, measured by IVCF-20, at baseline and at the end of the study. After the intervention, the number of frail individuals swelled in both groups. However, the IG presented a lower percentage (21.7%) of frail individuals than the CG (RR = 0.74; 95%CI = 0.60-0.91), demonstrating an intervention efficacy of 26%.

**Table 2:** Stratification of the older adults by the clinical-functional vulnerability index (IVCF-20) at baseline and at the end of the study, Montes Claros, Minas Gerais, Brazil, 2018.

Stratification of clinical-functional vulnerability by IVCF-20	Baseline			End line			
	Intervention (n = 197) n (%)	Control (n = 205) n (%)	p	Intervention (n = 197) n (%)	Control (n = 205) n (%)	p	RR (95%CI)
Frail	40 (20.2)	51 (24.9)	0.057	42 (21.7)	60 (29.3)	0.004	0.74 (0.60–0.91)
Not frail	157 (79.8)	154 (75.1)		155 (78.3)	145 (70.7)		1

RR: relative risk; 95%CI: 95% confidence interval.

The comparison of IVCF-20 variables between the groups at baseline and at the end of the study is presented in Table 3. At baseline, the groups were homogeneous in relation to the items “self-perception of positive health,” “independence for instrumental and basic daily living activities (ADL),” “mood,” “body mass index (BMI) ≥ 22,” “gait speed ≤ 5,” “no difficulty walking,” “no hearing problem,” and “polypathology” (presence of five or more diseases). The groups were not homogeneous regarding “cognition,” “no change of reach, grip and pincer,” “no weight loss,” “calf circumference ≥ 31,” “without two or more falls in the last year,” “no urinary incontinence,” “no vision problem,” “no polypharmacy” (use of five or more medications), and “no hospitalization in the last six months.” After intervention, there was an increase in the proportion of independent older adults for instrumental ADL in the intervention group, with a statistically significant difference in relation to the control group (RR = 1.16; 95%CI = 1.07-1.26).

**Table 3:** Characteristics of the clinical-functional vulnerability index (IVCF-20) in the intervention and control groups, at baseline and at the end of the study, Montes Claros, Minas Gerais, Brazil, 2018.

IVCF – 20 Characteristic	Baseline			End of the study			
	Intervention (n = 197)	Control (n = 205)	p	Intervention (n = 197)	Control (n = 205)	p	RR (95%CI)
	n (%)	n (%)		n (%)	n (%)		
1. Positive self-perceived health	146 (74.6)	148 (72.1)	0.361	123 (62.9)	139 (67.8)	0.084	-
2. Independent activity of daily living (ADL)							
Instrumental ADL	131 (66.8)	147 (71.7)	0.077	139 (70.7)	125 (61.0)	< 0.001	1.16 (1.07–1.26)
Basic ADL	179 (90.6)	192 (93.7)	0.052	174 (88.3)	186 (90.7)	0.189	-
3. Cognition							
Did any relative or friend say you are forgotten? No	108 (55.1)	130 (63.4)	0.004	114 (58.2)	130 (63.4)	0.072	-
Did your forgetfulness get worse in the last months? No	146 (74.4)	168 (82.0)	0.002	181 (91.6)	161 (78.6)	< 0.001	1.17 (1.11–1.22)
Is forgetfulness preventing you to perform any daily activity? No	164 (83.4)	186 (90.7)	< 0.001	184 (93.2)	179 (87.3)	0.001	1.07 (1.03–1.11)
4.Mood							
Have you been feeling sadness, discouragement or hopelessness in the last month? No	122 (62.1)	124 (60.5)	0.591	131 (67.0)	124 (60.5)	0.025	1.11 (1.01–1.21)
Have you lost interest or pleasure in the activities in the last month? No	158 (80.2)	155 (75.6)	0.060	172 (87.3)	150 (73.1)	< 0.001	1.19 (1.13–1.27)
5. Mobility							
There was no change in range, grip and pincer	192 (97.3)	189 (92.3)	< 0.001	192 (97.3)	189 (92.3)	< 0.001	1.05 (1.03–1.08)
No weight loss	188 (95.3)	180 (87.8)	0.001	178 (89.8)	174 (84.9)	0.013	1.06 (1.01–1.11)
BMI ≥ 22	166 (84.0)	166 (81.0)	0.190	163 (82.4)	163 (79.5)	0.221	-
Calf circumference ≥ 31	185 (93.9)	180 (87.8)	< 0.001	186 (94.7)	187 (91.2)	0.021	1.04 (1.01–1.07)
Gait speed ≤ 5 seconds	139 (69.7)	149 (72.6)	0.274	107 (52.5)	117 (57.1)	0.127	-
No difficulty to walk	159 (80.5)	162 (79.1)	0.559	155 (78.1)	150 (73.1)	0.051	-
Did not presented two or more falls in the past year	165 (84.0)	182 (88.7)	0.019	160 (81.2)	159 (77.6)	0.138	-
No sphincter incontinence	174 (88.3)	165 (80.5)	< 0.001	148 (75.1)	130 (63.4)	< 0.001	1.16 (1.08–1.26)
6. Communication							
No vision impairment	176 (89.8)	164 (80.0)	< 0.001	154 (76.2)	138 (67.3)	0.001	1.14 (1.06–1.28)
No hearing impairment	186 (94.5)	195 (95.2)	0.684	169 (84.8)	175 (85.3)	0.785	-
7. Multiple comorbidities							
Polypharmacy? No	138 (69.9)	127 (62.0)	0.005	133 (67.6)	132 (64.4)	0.263	-
Politapathology? No	132 (66.4)	126 (61.5)	0.088	168 (85.9)	143 (69.7)	< 0.001	1.23 (1.16–1.31)
Hospitalization in the past six months? No	191 (96.9)	189 (92.3)	0.001	181 (92.0)	187 (91.2)	0.615	-

RR: relative risk; 95%CI: 95% confidence interval.

Regarding cognition, the older adults in the intervention group also presented more positive responses than those of the CG for the items “forgetfulness has not worsened in the recent months” (RR = 1.17; 95%CI = 1.11-1.22) and “forgetfulness did not prevent daily activities” (RR = 1.07; 95%CI = 1.03-1.11). On mood, older adults in the intervention group presented higher percentages for “no manifestation of sadness, discouragement or hopelessness” (RR = 1.11; 95%CI = 1.01-1.21) and also “had no loss of interest or pleasure” (RR = 1.19; 95%CI = 1.13-1.27). In the mobility item, most older adults in the IG “did not present alteration of reach, grip, and pincer” (RR = 1.05; 95%CI = 1.03-1.08), “had no weight loss” (RR = 1.06; 95%CI = 1.01-1.11), maintained the “calf circumference ≥ 31” (RR = 1.04; 95%CI =1.01-1.07) and they did not have “urinary incontinence” (RR = 1.16; 95%CI = 1.08-1.26). Among the variables of the item communication, “no vision problem” was more frequent among the older adults in the IG, also with a statistically significant difference (RR = 1.14; 95%CI = 1.06-1.28). In the dimension of multiple comorbidities, there was a favorable difference for the IG in the item “without polypathologies” (RR = 1.23 (95%CI = 1.16-1.31).

Some items did not show changes after the intervention: “self-perceived positive health,” “independence for basic ADL,” “no observation of any family member or friend about forgetfulness,” “walking speed ≤ 5,” “no difficult to walk,” “without two or more falls in the last year,” “no hearing problems,” “no polypharmacy,” and “no hospitalization in the last six months.”

## DISCUSSION

The results of the study showed that the effect of health matrix actions for older adults assisted by FHS teams on the dimensions of frailty measured with the IVCF-20 was positive, with an improvement in the percentages for most items and dimensions assessed. The percentage of frailty in the group whose professionals participated in the MS, at the end of the study, was lower than that of the control group. Thus, we emphasize that the implementation of care models supporting the identification and management of chronic conditions, such as frailty syndrome, can contribute to the improvement and/or maintenance of the clinical-functional stratum of older adults in the FHS^13^. The development of pedagogical and clinical-care technical actions by health professionals can improve or delay negative repercussions on the functionality and quality of life of the older adults^1,2,4,7^.

The results found are in accordance with the literature, that attests to the need for early identification of frailty as an essential point for understanding its dynamic and unpredictable evolution in the course of long-term health, emphasizing the benefit of timely interventions in order to reduce the individual and collective burden^28^. Frail older adults present reduction of homeostatic reserve and/or ability to adapt to injuries, being predisposed to greater vulnerability and functional impairment^13,23,24,28,30,31^. The screening of the different strata of clinical and functional vulnerability in the older adults of PHC, with the IVCF, was the starting point for the planning of the matrix support of this research.

Regarding the interviewees’ data, in the baseline, “age,” “sex,” and “income” were the variables that showed similarities and comparability between the control and intervention groups. At the end of the intervention, there was an increase in the percentage of instrumental ADL of older adults in the IG, in addition to improving the responses in the cognition and mood dimensions. These results can be explained by the improvement of the functionality in older adults due to a possible approach of the variables mood and cognition, with favorable reflexes in their independence and reduction of vulnerability. The literature correlates the mental domain, physical capacity, and frailty, mentioning risks and consequences of this association regarding the worsening of the individual’s health and quality of life^7,15,22^. Furthermore, promoting actions to preserve the autonomy and functional capacity of older adults reduces the adverse outcomes of frailty, because functional loss is always pathological and requires careful investigation^1,4,7,9,10,14,15,22^.

There was also an improvement in the intervention group in relation to the control group of the percentage in topics of health determinants of the IVCF-20: 1) mobility (“did not present alteration of reach, grip and pincer,” “had no weight loss,” “calf circumference ≥ 31,” and “no urinary incontinence”); 2) communication (“has no vision problem”); and 3) multiple comorbidities (“did not present polypathologies”). Notably, these dimensions were already significantly better in the intervention before training, however it is emphasized that they remain statistically significant at the end. It is also noteworthy the need for accurate investigation of mobility issues and in the evaluation of the older adults, especially incontinence^15,24,32^.

Therefore, these indicators complement the IVCF-20 in the screening of clinical and functional vulnerability of the older adults and the identification of frailty, which represents a dynamic, multifactorial clinical state resulting from the sum of deficits^15,16,24,25,31^ that usually culminate in hostile outcomes to affected individuals and to the public system^2,4,7,15,23,25,32^. Studies show the association of frailty with reduced mobility, weight loss^15,30,31^, presence of polypathologies^15,24,25,32^, incontinence and impaired vision, in addition to other geriatric syndromes^15,30,31^.

It is not possible to evaluate how the MS separately influenced each of the dimensions measured by the IVCF-20. Although these relationships are extensively discussed in the literature, the aim of study was to evaluate possible changes in the frailty indicator, which were restricted to some aspects (instrumental activities and cognition), but not to all components of the frailty index used. Overall, the intervention may have contributed to the recognition of vulnerability strata, providing opportunities for sensitization and decision-making based on care measures centered on this age group, capable of improving the functionality of the older adults in the IG.

The essence of this work was the approximation between primary care professionals (PHC teams) and secondary care (geriatrics of the SUS). The sensitization of professionals of the FHS and the co-management of frailty syndrome reinforce the implementation of integrated, proactive, and people-centered care models that promote assertive intersectoral strategies, capable of predicting and delaying negative outcomes for older adults, family members/caregivers, and the public system^8^.

The results should be observed based on some limitations. Frailty syndrome is a dynamic and multifactorial condition, with potential transitions between natural regression or worsening. Another issue that limits the study is the lack of a standardized and universal tool for identifying frailty. Similarly, memory bias is another aspect to be considered, since some variables were measured based on the report of the older adults or family members. Also, it should be considered that the development of matrix support activities in medical residency fields defines an unusual situation, which may have influenced the results by greater professional support.

Despite the limitations observed, the study indicates to positive effects for an auxiliary model of MS for the FHS teams in the health care of older adults. The intervention proved to be effective and safe in the awareness of PHC professionals regarding the particularities of the health of older adults. The intervention was also promising in establishing a collective environment that respects the interprofessional relationship in a network. In the current scenario, longevity represents achievements of the contemporary society, but it also exposes profound changes in the epidemiological profile of health conditions, which require bold strategies of individual and collective care^1,4^. Thus, it is expected that the results of this study can support the future implementation of this intervention in public and supplementary services in order to contribute positively to the co-management of comprehensive care for the older adults.

It is also important to highlight that the Brazilian Ministry of Health considered, in the evaluation of the quality of primary care, the standard related to the matrix support received by the family health team as a strategy of permanent education of its workers^17,18^. It is also important for managers to provide this type of support to teams, in order to better qualify and fix the FHS professionals^17,19^. In this study, the actions of matrix support in the health of the older adults in PHC reduced the clinical and functional vulnerability of people aged 60 years or older. The intervention by training FHS professionals was also effective in the care of non-frail older adults registered and assisted by these teams.

Therefore, the MS – as pedagogical attribution and horizontal care for health teams – has the potential to contribute to the articulation of care models, with positive results for the health of older adults. Also, it works as a relational instrument, enabling the approximation between the levels of care with the improvement and strengthening of the principles and guidelines of the SUS.

## Footnotes

a Available from: http://www.ensaiosclinicos.gov.br/rg/RBR-7b9xff/
