# Supplementary material for: Impact of matrix support on older adults in primary care: randomized community trial
Source: Rev Saude Publica. 2021 Apr 5;55:10. doi: 10.11606/s1518-8787.2021055002685 (PMC8023320; doi:10.11606/s1518-8787.2021055002685)
Supplement: Translation

# Translation: Impacto do apoio matricial a idosos na atenção primária: ensaio comunitário randomizado

**Authors:** Luciana Colares Maia, Thomaz de Figueiredo Braga Colares, Edgar Nunes de Morais, Simone de Melo Costa, Antônio Prates Caldeira

**Affiliations:** I Brasil Universidade Estadual de Montes Claros. Centro Mais Vida Eny Faria de Oliveira. Departamento de Clínica Médica. Montes Claros, MG, Brasil; II Brasil Universidade Estadual de Montes Claros. Centro Mais Vida Eny Faria de Oliveira. Montes Claros. MG, Brasil; III Brasil Universidade Federal de Minas Gerais. Instituto Jenny Faria de Oliveira. Núcleo de Geriatria e Gerontologia. Belo Horizonte, MG, Brasil; IV Brasil Universidade Estadual de Montes Claros. Centro de Ciências Biológicas e da Saúde. Departamento de Odontologia. Montes Claros, MG, Brasil; V Brasil Universidade Estadual de Montes Claros. Centro de Ciências Biológicas e da Saúde. Departamento de Saúde da Mulher e da Criança. Montes Claros, MG, Brasil

**Keywords:** Saúde do Idoso, Apoio Matricial, Atenção Primária à Saúde, Saúde Pública, Ensaio Clínico

## Abstract

**OBJETIVO:**

Analisar o impacto do apoio matricial em saúde ao idoso na atenção primária de acordo com as dimensões de fragilidade aferidas por meio do índice de vulnerabilidade clínico-funcional (IVCF-20).

**MÉTODOS:**

Trata-se de ensaio comunitário randomizado controlado (ECRC), desenvolvido no norte de Minas Gerais, Brasil, em 2018. Inicialmente ocorreu, por meio do IVCF-20, a estratificação de vulnerabilidade clínico-funcional dos idosos assistidos por seis equipes de Estratégia de Saúde da Família. Posteriormente, três equipes foram sorteadas para receber apoio matricial durante seis meses, e as demais, para controle. Nesta intervenção, desenvolveram-se atividades educativas presenciais para as equipes de saúde. Realizou-se estatística descritiva, seguida de análise bivariada pelo teste qui-quadrado de Pearson, para comparação das variáveis do IVCF-20 entre os dois momentos (antes e após a intervenção), com nível de significância de 5%. Estimaram-se os riscos relativos (RR) e os respectivos intervalos de confiança de 95% (IC95%).

**RESULTADOS:**

Os grupos eram similares antes da intervenção, e o efeito de ações de matriciamento foi positivo para a maioria das dimensões aferidas pelo IVCF-20 (atividade de vida diária instrumental, cognição, humor, mobilidade, comunicação e comorbidades múltiplas). O percentual de fragilidade no grupo assistido por profissionais participantes do apoio matricial, ao final da pesquisa, foi inferior ao do grupo controle.

**CONCLUSÕES:**

As ações de matriciamento, como atribuição pedagógica e assistência horizontal para as equipes de saúde, têm o potencial de contribuir para a articulação de modelos de cuidado para a saúde do idoso.

**REBEC:**

registro BR-7b9xff

## INTRODUÇÃO

O rápido envelhecimento da população brasileira define um perfil demográfico e epidemiológico com crescentes e exigentes demandas ao sistema de saúde, fazendo-se necessária a articulação de ferramentas para organização, estruturação e integração dos diferentes pontos de atenção à pessoa idosa^1^. Esse fenômeno requer modelo assistencial que aborde o idoso de forma integral, em concordância com princípios e diretrizes do Sistema Único de Saúde (SUS)^1,2,5^. O grande desafio consiste em remodelar o sistema de saúde fragmentado em uma rede de atenção à saúde que responda oportunamente de forma proativa, contínua e integrada, baseada nos aspectos demográficos e epidemiológicos da população^6,7^.

A estrutura ideal de atenção à saúde deve direcionar o fluxo e os serviços existentes nas redes de atenção, agindo de forma cooperativa, interdependente e coordenado pela Atenção Primária à Saúde (APS)^1,2,7,8^. Nesse contexto, a Estratégia de Saúde da Família (ESF), que constitui a forma prioritária de organização da APS no país, tem destaque na operacionalização da rede de atendimento à população idosa^1,7^. A atenção integral pressupõe identificar os mais vulneráveis, assim como acompanhar a evolução clínico-funcional e o fluxo na rede organizada^9^. Isso resultaria em menos consultas e internações hospitalares e melhor gerenciamento da funcionalidade, além de melhor relação custo/efetividade no sistema de saúde^1,6,7,10,11,13,14^.

Existem ricas experiências de fortalecimento da APS que combinam empoderamento das equipes de saúde com apoio à gestão dos cuidados em saúde, como o matriciamento^17,18^. O apoio matricial (AM) em saúde surgiu como estratégia inovadora, fundamentada na promoção de um sistema organizacional de rede de serviços assistenciais e educacionais contínuos no âmbito do SUS^17^. A ideia principal do AM é operacionalizar o funcionamento integrado da rede por meio da relação interprofissional ecoparticipativa de seus elementos constitutivos^17,19-21^. O matriciamento consiste na reconstrução teórica e conceitual da metodologia de trabalho interdisciplinar em saúde, que considera a relação entre equipes de referência (ESF) e profissionais de apoio matricial (atenção secundária). O apoio depende da relação entre equipes de saúde, do alargamento de cenários de atenção especializada e da elaboração compartilhada, entre profissionais da equipe de referência e especialistas que ofertam o AM, de diretrizes clínicas e sanitárias^20^. Portanto, os elementos constitutivos do AM envolvem serviços que compõem a rede SUS e relações conformadas entre equipes para operacionalizar o referido apoio.

Entre as experiências exitosas de AM, destaca-se o caso da saúde mental, com modelo reformador do cuidado em práticas interprofissionais compartilhadas associadas à corresponsabilidade entre equipe e usuários, contribuindo para o desenvolvimento de ações de descentralização da assistência^17,18^. A proposta de modelo matricial na atenção à saúde do idoso é uma ideia ainda não consolidada no país e está em consonância com as necessidades dos serviços de saúde para potencializar a rede de atenção^1,3,8^.

O encaminhamento para os diferentes níveis de assistência da rede de atenção à saúde da pessoa idosa varia de acordo com o grau de vulnerabilidade identificado (frágil e não frágil)^1,7,10,12,15,16,22^. Para o rastreio dos estratos clínico-funcionais nessa população, existem diversosinstrumentos^15,16,22,26^, incluindo o índice de vulnerabilidade clínico-funcional (IVCF-20), que foi desenvolvido e validado no Brasil e tem boa correlação com outros instrumentos utilizados internacionalmente^15,16,26,27^. Pela facilidade de aplicação, o IVCF-20 é uma boa ferramenta para as equipes da ESF na avaliação e no seguimento dos idosos^15,16^. O presente estudo objetivou analisar o impacto do apoio matricial em saúde a idosos assistidos pela ESF a partir da aferição do nível de fragilidade, segundo o IVCF-20.

## MÉTODOS

Trata-se de ensaio comunitário randomizado controlado (ECRC), realizado no município de Montes Claros, na região Sudeste do Brasil, em 2018. Na época do estudo, a população urbana era de aproximadamente 400 mil habitantes e registrava mais de 80% de cobertura pela ESF, com 121 equipes. Considerando a necessidade de contextos com baixa rotatividade entre os profissionais de saúde, a pesquisa foi desenvolvida em polos de abrangência do Programa de Residência Médica em Medicina de Família e Comunidade (PRMMFC). No período, o município tinha 12 polos regionais, compostos cada um por três equipes, e cada equipe era responsável por cinco microáreas. Foram sorteados dois polos, entre os 12, para imediata intervenção, e dois polos para grupo controle, utilizando-se o programa gerador de números aleatórios (randomnumbergeneration.intemodino.com). Os polos sorteados apresentavam semelhanças em relação à composição das equipes, eram áreas de abrangência de PRMMFC e apresentavam similaridade sociodemográfica. A definição das equipes para intervenção ou controle foi randomizada, por sorteio simples. A unidade de intervenção foi a equipe de saúde, e o desfecho foi aferido na população de idosos assistidos pelas respectivas equipes.

Para o cálculo amostral do número de idosos acompanhados em cada grupo, considerou-se o erro tipo 1 (alfa) de 5%, e o poder do estudo de 80% em um teste bicaudal. Foi considerada a taxa de vulnerabilidade clínico-funcional (fragilidade) de 25% e, após o matriciamento, uma taxa de 10%, ou seja, redução de 15%. Assim, a amostra proposta foi definida em, pelo menos, 150 idosos em cada grupo do estudo: intervenção e controle.

A partir da seleção aleatória das equipes de saúde, a inclusão dos idosos foi realizada também de forma aleatória, alicerçada na relação das pessoas com idade de 60 anos ou mais assistidas pelas equipes da Residência de Medicina de Família e Comunidade, respeitando critérios de elegibilidade para o estudo. Participaram da pesquisa idosos com capacidade de responder os questionários e, quando incapacitados, aqueles que tinham cuidador/responsável disponível durante a visita para fornecer as informações solicitadas. A coleta foi realizada no domicílio, nos turnos matutino, vespertino ou noturno e em todos os dias da semana, preferencialmente com agendamento prévio. Idosos não localizados em pelo menos três visitas, em dias e horários diferentes, foram substituídos por sorteio.

O diagrama Consort (*Consolidated Standards of ReportingTrials)* (Figura 1) apresenta o detalhamento da inclusão, alocação e seguimento dos participantes do estudo, no grupo intervenção (com apoio matricial às ESF) e no grupo controle (sem apoio matricial às ESF). Os 402 participantes elegíveis para compor a amostra de idosos foram distribuídos em: 197 no grupo intervenção (GI) e 205 no grupo controle (GC). O número maior de idosos em relação ao cálculo amostral foi incluído levando em conta a possibilidade de perdas, o que não ocorreu ao longo do período de estudo.

**Figura:** Diagrama Consort de alocação, seguimento e análise dos indivíduos do apoio matricial em saúde do idoso, Montes Claros, Minas Gerais, Brasil.

Os idosos selecionados responderam ao IVCF-20, um questionário de triagem com caráter multidimensional, de rápida aplicação (cinco a 10 minutos) e de fácil interpretação do resultado, que pode ser realizado por todos da equipe de saúde, o que, provavelmente, contribuiu para resultados positivos no contexto do reconhecimento e gerenciamento multiprofissional da fragilidade na APS^15,16,26^.

Esse instrumento engloba domínios considerados preditores de vulnerabilidade clínico-funcional e desfechos adversos, como declínio funcional e óbito em pessoas idosas^15,16,26,27^. Ademais, considera as seguintes dimensões: autopercepção de saúde, atividades de vida diária (AVD), cognição, humor, mobilidade, comunicação e comorbidades múltiplas. O IVCF-20 proporciona a criação de um escore que define o risco de fragilidade. O escore final para estratificar a fragilidade no presente estudo foi dicotomizado em frágil (escore ≥ 15) e não frágil (escore < 15)^15,16,22,26,27^. Além do IVCF-20, foram coletadas informações sobre idade, sexo e renda dos idosos participantes.

A coleta de dados foi realizada por equipe especialmente treinada, composta por profissionais da enfermagem e estudantes de medicina. Previamente ao início da pesquisa, foi conduzido um projeto piloto, em área distinta daquela selecionada para a pesquisa, para a calibração dos entrevistadores. A coleta de dados ocorreu antes (linha de base) e seis meses após o término da intervenção (linha final) por meio da capacitação dos profissionais da ESF para apoio matricial à saúde dos idosos. Os entrevistadores foram os mesmos, tanto no momento pré-intervenção como no pós-intervenção, e não tinham conhecimento de quais equipes de saúde estariam sob intervenção ou controle.

A capacitação das equipes de saúde foi realizada durante seis meses nos polos do PRMMFC, com encontros mensais coordenados por médica geriatra atuante no SUS. Instituiu-se cronograma de capacitação modular norteada pelos eixos estruturantes da saúde do idoso^7^, sem prejuízo para o serviço das unidades de saúde selecionadas para intervenção. A equipe recebia antecipadamente a convocação pela Secretaria Municipal de Saúde (SMS) e, por telefone, a confirmação do encontro pela responsável do AM. Participaram das atividades educativas os profissionais integrantes de cada polo: três da medicina, seis da enfermagem e quinze agentes comunitários de saúde. Foram considerados elegíveis para participação da intervenção todos os profissionais das categorias descritas anteriormente, vinculados aos polos sorteados. A frequência de participação foi de 100%.

Essa proposta foi norteada pelos moldes do matriciamento da saúde mental, já realizado em toda a APS do munícipio. Assim, cada reunião mensal tinha a duração de quatro horas, fundamentada em ações tutoriais, aula dialogada, discussão de casos clínicos, além de atendimento e construção de planos terapêuticos de forma conjunta. Ressalta-se que os planejamentos educativo e assistencial dos encontros valorizavam as demandas das equipes, embasando-se em discussão de casos clínicos de idosos com a estratificação clínico-funcional, seguida de elaboração e acompanhamento compartilhado de planos de cuidados, com o gerenciamento da saúde do usuário pelas equipes da APS.

Em um primeiro momento, realizou-se enquete sobre os principais problemas apresentados pelos idosos do GI: esquecimento, quedas, perda da funcionalidade, polifarmácia, iatrogenia, insuficiência familiar e incontinência urinária. Os temas agregavam eixos estruturantes do cuidado integral de idosos (gigantes da geriatria)^7^– 1) síndrome de fragilidade; 2) incapacidade cognitiva; 3) instabilidade postural, quedas e imobilidade; 4) iatrogenia; e 5) incontinência –, tópicos que constituem as dimensões do IVCF-20^27^. Assim, foi realizada a programação mensal da experiência matricial em saúde do idoso, com respeito às demandas das equipes. Reitera-se a disponibilização do *e-mail* e telefone da tutora para o GI.

Realizou-se estatística descritiva, seguida de análise bivariada pelo teste qui-quadrado de Pearson para comparação das variáveis do IVCF-20antes e após a intervenção, assumindo-se o nível de significância de 5%. O risco relativo (RR) foi estimado para a classificação geral do índice de fragilidade a partir dos escores finais do instrumento (categorizados em frágeis e não frágeis) e também para as variáveis que compõem as dimensões do IVCF-20. Os dados foram processados por meio do *software* IBM^®^SPSS^®^, versão 22.0.

Este estudo foi conduzido de acordo com a Resolução 466/12 do Conselho Nacional de Saúde, Ministério da Saúde, com o parecer nº 1.628.652. Também foi registrado na Plataforma de Registro Brasileiro de Ensaios Clínicos (ReBEC)^a^, sob o número de registro BR-7b9xff.

Todos os participantes foram devidamente informados sobre a pesquisa e apresentaram sua aquiescência por meio do termo de consentimento livre e esclarecido (TCLE). Para aqueles que não sabiam assinar, realizou-se coleta de digitais em campo específico do TCLE. Garantiram-se anonimato e confidencialidade das informações fornecidas, utilizadas exclusivamente para fins da pesquisa.

Ressalta-se que, com a conclusão da coleta de dados pós-intervenção, as demais equipes de saúde receberam a mesma intervenção educativa. Ao longo do processo de pesquisa, os idosos reconhecidos com elevado grau de fragilidade foram encaminhados para acompanhamento específico (atenção secundária). No decurso do estudo, os idosos do GC continuaram sendo assistidos e acompanhados pelas equipes da ESF a que estavam adstritos, utilizando os recursos habituais disponíveis no município.

## RESULTADOS

A Tabela 1 apresenta a comparação de algumas características dos GI e GC na linha de base do estudo. Percebeu-se que a maioria dos idosos era do sexo feminino, tinha idade entre 60 a 74 anos e renda de até dois salários vigentes no período da coleta de dados desta pesquisa. Essas variáveis foram definidas para análise de comparabilidade dos grupos, não se registrando diferenças estatisticamente significantes entre as variáveis demográficas e econômicas (sexo, idade e renda) dos grupos, na linha de base do trabalho.

**Tabela 1:** Características demográficas e econômicas dos grupos intervenção (GI) e controle (GC), na linha de base do estudo, Montes Claros, Minas Gerais, Brasil, 2018.

Variável	Intervenção (GI)	Controle (GC)	P
	n (%)	n (%)	
Sexo			0,224
Feminino	126 (63,9)	138 (67,3)	
Masculino	71 (36,1)	67 (32,7)	
Idade (anos)			0,916
60–74	118 (58,3)	118 (57,6)	
75–84	56 (29,4)	62 (30,2)	
≥ 85	23 (12,3)	25 (12,2)	
Renda (em salários-mínimos)^a^			0,202
> 2	74 (42,8)	91 (46,7)	
≤ 2	109 (57,2)	104 (53,3)	

^a^ Salário mínimo vigente em 2018 (R$ 937,00).

A Tabela 2 apresenta a estratificação dos idosos quanto à vulnerabilidade clínico-funcional, mensurada pelo IVCF-20, na linha de base e ao final do estudo.Após a intervenção, o número de frágeis elevou-se nos dois grupos. No entanto, o GI apresentou menor percentual (21,7%) de idosos frágeis que o GC (RR = 0,74; IC95% = 0,60–0,91), demonstrando uma eficácia da intervenção em 26%.

**Tabela 2:** Estratificação dos idosos pelo índice de vulnerabilidade clínico-funcional (IVCF-20) na linha de base e ao final do estudo, Montes Claros, Minas Gerais, Brasil, 2018.

Estratificação da vulnerabilidade clínico-funcional pelo IVCF-20	Linha de base			Linha final			
	Intervenção (n = 197) n (%)	Controle (n = 205) n (%)	p	Intervenção (n = 197) n (%)	Controle (n = 205) n (%)	p	RR (IC95%)
Frágil	40 (20,2)	51 (24,9)	0,057	42 (21,7)	60 (29,3)	0,004	0,74 (0,60–0,91)
Não frágil	157 (79,8)	154 (75,1)		155 (78,3)	145 (70,7)		1

RR: risco relativo; IC95%: intervalo de confiança de 95%.

A comparação das variáveis do IVCF-20 entre os grupos na linha de base e ao final do estudo encontra-se na Tabela 3. Na linha de base, os grupos eram homogêneos em relação aos itens “autopercepção da saúde positiva”, “independência para as atividades de vida diária (AVD) instrumental e básica”, “humor”, “índice de massa corporal (IMC) ≥ 22”, “velocidade de marcha ≤ 5”, “sem dificuldade para caminhar”, “sem problema de audição” e “sempolipatologia” (presença de cinco ou mais doenças). Os grupos não se mostraram homogêneos em relação a “cognição”, “sem alteração de alcance, preensão e pinça”, “sem perda de peso”, “circunferência da panturrilha ≥ 31”, “sem duas ou mais quedas no último ano”, “sem incontinência urinária”, “sem problema de visão”, “sem polifarmácia” (uso de cinco ou mais medicamentos) e “sem internação nos últimos seis meses”. Após intervenção, verificou-se aumento da proporção de idosos independentes para as AVD instrumental no GI, com diferença estatisticamente significante em relação ao GC (RR = 1,16; IC95% = 1,07–1,26).

**Tabela 3:** Variáveis do índice de vulnerabilidade clínico-funcional (IVCF-20) nos grupos intervenção e controle, na linha de base e final do estudo, Montes Claros, Minas Gerais, Brasil, 2018.

IVCF – 20	Linha de base			Final do estudo			
	Intervenção (n = 197)	Controle (n = 205)	p	Intervenção (n = 197)	Controle (n = 205)	p	RR (IC95%)
	n (%)	n (%)		n (%)	n (%)		
1. Autopercepção de saúde positiva	146 (74,6)	148 (72,1)	0,361	123 (62,9)	139 (67,8)	0,084	-
2. Atividade de vida diária (AVD) independente							
AVD instrumental	131 (66,8)	147 (71,7)	0,077	139 (70,7)	125 (61,0)	< 0,001	1,16 (1,07–1,26)
AVD básica	179 (90,6)	192 (93,7)	0,052	174 (88,3)	186 (90,7)	0,189	-
3. Cognição							
Algum familiar ou amigo falou que você está esquecido? Não	108 (55,1)	130 (63,4)	0,004	114 (58,2)	130 (63,4)	0,072	-
Esquecimento piorando nos últimos meses? Não	146 (74,4)	168 (82,0)	0,002	181 (91,6)	161 (78,6)	< 0,001	1,17 (1,11–1,22)
Esquecimento está impedindo a realização de alguma atividade do cotidiano? Não	164 (83,4)	186 (90,7)	< 0,001	184 (93,2)	179 (87,3)	0,001	1,07 (1,03–1,11)
4.Humor							
No último mês você ficou com tristeza, desânimo ou desesperança? Não	122 (62,1)	124 (60,5)	0,591	131 (67,0)	124 (60,5)	0,025	1,11 (1,01–1,21)
No último mês você perdeu o interesse ou prazer nas atividades? Não	158 (80,2)	155 (75,6)	0,060	172 (87,3)	150 (73,1)	< 0,001	1,19 (1,13–1,27)
5. Mobilidade							
Não teve alteração de alcance, preensão e pinça	192 (97,3)	189 (92,3)	< 0,001	192 (97,3)	189 (92,3)	< 0,001	1,05 (1,03–1,08)
Não teve perda de peso	188 (95,3)	180 (87,8)	0,001	178 (89,8)	174 (84,9)	0,013	1,06 (1,01–1,11)
IMC ≥ 22	166 (84,0)	166 (81,0)	0,190	163 (82,4)	163 (79,5)	0,221	-
Circunferência da panturrilha ≥ 31	185 (93,9)	180 (87,8)	< 0,001	186 (94,7)	187 (91,2)	0,021	1,04 (1.01–1,07)
Velocidade de marcha ≤ 5 segundos	139 (69,7)	149 (72,6)	0,274	107 (52,5)	117 (57,1)	0,127	-
Sem dificuldade para caminhar	159 (80,5)	162 (79,1)	0,559	155 (78,1)	150 (73,1)	0,051	-
Não teve duas ou mais quedas no último ano	165 (84,0)	182 (88,7)	0,019	160 (81,2)	159 (77,6)	0,138	-
Sem incontinência esfincteriana	174 (88,3)	165 (80,5)	< 0,001	148 (75,1)	130 (63,4)	< 0,001	1,16 (1,08–1,26)
6. Comunicação							
Sem problema de visão	176 (89,8)	164 (80,0)	< 0,001	154 (76,2)	138 (67,3)	0,001	1,14 (1,06–1,28)
Sem problema de audição	186 (94,5)	195 (95,2)	0,684	169 (84,8)	175 (85,3)	0,785	-
7. Comorbidades múltiplas							
Polifarmácia? Não	138 (69,9)	127 (62,0)	0,005	133 (67,6)	132 (64,4)	0,263	-
Politapatologia? Não	132 (66,4)	126 (61,5)	0,088	168 (85,9)	143 (69,7)	< 0,001	1,23 (1,16–1,31)
Internação nos últimos seis meses? Não	191 (96,9)	189 (92,3)	0,001	181 (92,0)	187 (91,2)	0,615	-

RR: risco relativo; IC95%: intervalo de confiança de 95%.

Em relação à cognição, os idosos do GI também apresentaram respostas mais positivas que os do GC para os itens “esquecimento não piorou nos últimos meses” (RR = 1,17; IC95% = 1,11–1,22) e “esquecimento não impediu a realização de atividades do cotidiano” (RR = 1,07; IC95% = 1.03–1,11). Sobre o humor, idosos no grupo de intervenção apresentaram maiores percentuais para “não manifestação de tristeza, desânimo ou desesperança” (RR = 1,11; IC95% = 1,01–1,21) e “não teve perda de interesse ou prazer” (RR = 1,19; IC95% = 1,13–1,27). No quesito mobilidade, a maioria dos idosos do GI “não apresentou alteração de alcance, preensão e pinça” (RR = 1,05; IC95% = 1,03–1,08), “não teve perda de peso” (RR = 1,06; IC95% = 1,01–1,11), manteve a “circunferência da panturrilha ≥ 31 (RR = 1,04; IC95% = 1,01–1,07) e não teve “incontinência urinária” (RR = 1,16; IC95% = 1,08–1,26). Entre as variáveis do item comunicação, “sem problema de visão” foi mais frequente entre idosos do GI, também com diferença estatisticamente significativa (RR = 1,14; IC95% = 1,06–1,28). Na dimensão de comorbidades múltiplas, registrou-se diferença favorável para o GI no item “sem polipatologias” (RR = 1,23 (IC95% = 1,16–1,31).

Alguns itens não mostraram alterações após a intervenção: “autopercepção de saúde positiva”, “independência para AVD básicas”, “não observação de algum familiar ou amigo sobre esquecimento”, “velocidade de marcha ≤ 5”, “sem dificuldade para caminhar”, “sem duas ou mais quedas no último ano”, “sem problemas de audição”, “sem polifarmácia” e “sem internação nos últimos seis meses”.

## DISCUSSÃO

Os resultados do estudo demonstraram que foi positivo o efeito de ações de matriciamento em saúde para idosos assistidos por equipes da ESF sobre as dimensões da fragilidade aferidas por meio do IVCF-20, com melhoria dos percentuais para a maioria dos itens e dimensões avaliados. O percentual de fragilidade no grupo cujos profissionais participaram do AM, ao final do estudo, foi inferior ao do GC. Reitera-se, assim, que a implementação de modelos de cuidado que apoiam o reconhecimento e o gerenciamento de condições crônicas, como a síndrome de fragilidade, pode colaborar com a melhora e/ou manutenção do estrato clínico-funcional dos idosos na ESF^13^. O desenvolvimento de ações técnicas pedagógicas e clínico-assistenciais por profissionais de saúde pode melhorar ou protelar repercussões negativas sobre a funcionalidade e a qualidade de vida da pessoa idosa^1,2,4,7^.

Os resultados encontrados são congruentes com a literatura, que atesta a necessidade da identificação precoce da fragilidade como ponto essencial para a compreensão de sua evolução dinâmica e imprevisível no curso da saúde em longo prazo, ressaltando o benefício de intervenções oportunas para a redução do ônus individual e coletivo^28^.Idosos frágeis apresentam redução da reserva homeostática e/ou da capacidade de adaptação aos agravos, sendo predispostos a maiores vulnerabilidade e prejuízo funcional^13,23,24,28,30,31^. O rastreio dos diferentes estratos de vulnerabilidade clínico-funcional nos idosos da APS, por meio do IVCF, foi o ponto de partida para o planejamento do AM desta pesquisa.

Em relação aos dados dos entrevistados, na linha de base, “idade”, “sexo” e “renda” foram as variáveis que demonstraram semelhanças e comparabilidade entre os grupos controle e intervenção. Ao final da intervenção, verificou-se aumento no percentual das AVD instrumentais dos idosos do GI, além de melhora das respostas nas dimensões cognição e humor. Esses resultados podem ser explicados pela melhora da funcionalidade dos idosos decorrente de possível abordagem das variáveis humor e cognição, com reflexos favoráveis em sua independência e redução da vulnerabilidade. A literatura faz correlação entre o domínio mental, capacidade física e fragilidade, mencionando riscos e consequências dessa associação quanto ao detrimento da saúde e qualidade de vida do individuo^7,15,22^. Ademais, promover ações para preservar a autonomia e a capacidade funcional do idoso reduz os desfechos adversos da fragilidade, pois a perda funcional é sempre patológica e requer investigação criteriosa^1,4,7,9,10,14,15,22^.

Observou-se ainda melhora no GI em relação ao GC do percentual em tópicos de determinantes de saúde do IVCF-20: 1) mobilidade (“não apresentou alteração de alcance, preensão e pinça”, “não teve perda de peso”, “circunferência da panturrilha ≥ 31” e “sem incontinência urinária”); 2) comunicação (“não possui problema de visão”); e 3) comorbidades múltiplas (“não apresentaram polipatologias”). Percebe-se que essas dimensões do instrumento já eram significativamente melhores na intervenção antes da capacitação, contudose ressalta que permanecem estatisticamente significantes ao final. Vale ressaltar ainda a necessidade de investigação acurada dos quesitos de mobilidade e na avalição dos idosos, especialmente a incontinência^15,24,32^.

Por conseguinte, esses indicadores complementam o IVCF-20 no *screening* da vulnerabilidade clínico-funcional dos idosos e da identificação da fragilidade, que representa um estado clínico dinâmico, multifatorial, resultante da somatória de déficits^15,16,24,25,31^ que geralmente culminam em desfechos hostis aos indivíduos acometidos e ao sistema público^2,4,7,15,23,25,32^. Os estudos demonstram a associação de fragilidade com mobilidade reduzida, perda de peso^15,30,31^, presença de polipatologias^15,24,25,32^, incontinência e comprometimento da visão, além de outras síndromes geriátricas^15,30,31^.

Não é possível avaliar de que forma o AM influenciou isoladamente cada uma das dimensões aferidas pelo IVCF-20. Embora essas relações sejam extensivamente discutidas na literatura, o objetivo do presente estudo foi avaliar possíveis mudanças no indicador de fragilidade, que ficaram restritas a alguns aspectos (atividades instrumentais e cognição), mas não a todos os componentes do índice de fragilidade utilizado. Globalmente, a intervenção pode ter contribuído para o reconhecimento dos estratos de vulnerabilidade, oportunizando a sensibilização e a tomada de decisão fundamentada em medidas de cuidado centradas nessa faixa etária, capazes de melhorar a funcionalidade dos idosos do GI.

A essência deste trabalho foi a aproximação entre os profissionais da atenção primária (equipes da APS) e secundária (geriatra do SUS). A sensibilização de profissionais da ESF e a cogestão da síndrome de fragilidade reforçam a implementação de modelos de assistência integrados, proativos e centrados nas pessoas, que promovam estratégias intersetoriais assertivas, capazes de prever e protelar desfechos negativos para idosos, familiares/cuidadores e o sistema público^8^.

Os resultados devem ser observados a partir de algumas limitações. A síndrome da fragilidade é uma condição dinâmica e multifatorial, com potenciais transições entre a regressão natural ou piora. Outra questão que limita o estudo é a inexistência de ferramenta padronizada e universal para detecção de fragilidade. Igualmente, o viés de memória é outro aspecto a ser considerado, já que algumas variáveis foram aferidas a partir do relato do próprio idoso ou de familiares. Deve-se considerar ainda que o desenvolvimento das atividades de AM em campos de residência médica define uma situação não usual, que pode ter influenciado os resultados pela maior adesão dos profissionais.

Apesar das limitações observadas, o estudo aponta efeitos positivos para um modelo auxiliar de AM às equipes da ESF na atenção à saúde do idoso. A intervenção mostrou-se efetiva e segura na sensibilização dos profissionais da APS quanto às particularidades da saúde da pessoa idosa. Também se revelou promissora ao instaurar um ambiente coletivo que respeite a relação interprofissional em rede. No cenário atual, a longevidade representa conquistas da sociedade contemporânea, contudo também expõe profundas mudanças no perfil epidemiológico das condições de saúde, que exigem estratégias ousadas de cuidado individual e coletivo^1,4^. Assim, espera-se que os resultados deste estudo possam subsidiar a implantação futura dessa intervenção em serviços públicos e suplementares com o intuito de contribuir positivamente na cogestão do cuidado integral aos idosos.

É relevante destacar ainda que o Ministério da Saúde considerou, na avaliação de qualidade da atenção básica, o padrão relacionado ao apoio matricial recebido pela equipe de saúde da família como estratégia de educação permanente de seus trabalhadores^17,18^. Salienta-se também a importância de os gestores propiciarem esse apoio às equipes, no sentido de melhor qualificar e fixar os profissionais de ESF^17,19^. No atual estudo, as ações de matriciamento em saúde do idoso na APS reduziram a vulnerabilidade clínico-funcional das pessoas com 60 anos ou mais. A intervenção pela capacitação de profissionais das ESF também se mostrou efetiva quanto aos cuidados dos idosos não frágeis cadastrados e assistidos pelas referidas equipes.

Portanto, o AM, como atribuição pedagógica e assistência horizontal para as equipes de saúde, tem o potencial de contribuir para a articulação de modelos de cuidado, com resultados positivos para a saúde do idoso. Igualmente, funciona como instrumento relacional, com potencial de aproximação entre os níveis de assistência por meio do aprimoramento e fortalecimento dos princípios e diretrizes do SUS.

## Footnotes

a Disponível em: http://www.ensaiosclinicos.gov.br/rg/RBR-7b9xff/
